# The Systemic Cytokine Environment Is Permanently Altered in Multiple Myeloma

**DOI:** 10.1371/journal.pone.0058504

**Published:** 2013-03-27

**Authors:** Mary M. Zheng, Zhifang Zhang, Kyle Bemis, Andrew R. Belch, Linda M. Pilarski, John E. Shively, Julia Kirshner

**Affiliations:** 1 Department of Biological Sciences, Purdue University, West Lafayette, Indiana, United States of America; 2 Division of Immunology, Beckman Research Institute of the City of Hope National Medical Center, Duarte, California, United States of America; 3 Department of Statistics, Purdue University, West Lafayette, Indiana, United States of America; 4 Department of Oncology, University of Alberta and Cross Cancer Institute, Edmonton, Alberta, Canada; 5 Department of Medical Oncology, University of Alberta and Cross Cancer Institute, Edmonton, Alberta, Canada; Istituto Superiore di Sanità, Italy

## Abstract

Multiple myeloma (MM) is an incurable bone marrow malignancy of the B cell lineage. Utilizing multiplex Luminex technology we measured levels of 25 cytokines in the plasma of normal donors (n = 177), those with monoclonal gammopathy of undetermined significance (n = 8), and MM patients (n = 55) with either active disease, on treatment, or in remission. The cytokine levels were compared between normal donors and MM patients as well as between various phases of MM, and discriminant analysis was used to create a predictive classification model based on the differentially expressed cytokines. Evaluating age- and gender-dependence of cytokine expression, we determined that with age there is a shift toward a pro-inflammatory environment. Moreover, we observed a strong gender bias in cytokine expression. However, the profile of differentially expressed cytokines was heavily skewed toward an anti-inflammatory, pro-tumorigenic response in patients with MM. Significantly, our predictive model placed all patients in remission in the same category as those with active disease. Thus, our study demonstrates that the homeostasis of systemic cytokines is not restored when MM patients enter remission, suggesting that once an individual has cancer, the microenvironment is permanently altered and the system is primed for a relapse.

## Introduction

Aging is a multifaceted phenomenon associated with multiple changes in the molecular, cellular, and physiological state of the organism. Over time various systems accumulate damage and can no longer adapt to further insults, and thus, aging has been linked to deteriorating health [1], [2]. Immunosenescence, a deterioration of the innate and adaptive immune responses as a result of reduced numbers of neutrophils, impaired phagocytosis, thymic atrophy, diminished T cell responses, compromised antigen presentation, and production of low affinity antibodies by B cells, is associated with increased morbidity and mortality from infections, autoimmune diseases, and various malignancies [3]. Another aspect of immunosenescence is deregulation of cytokine homeostasis resulting in the state “inflamm-aging”, or chronic systemic inflammation [4], [5]. This inflammation is sustained through a heightened secretion of pro-inflammatory cytokines, IL-1, IL-6, IL-15, IL-17, interferon alpha (IFNα, IFNγ, TNFα in individuals over 55 years of age [5], [6], [7], [8], [9], [10]. Supporting the systemic pro-inflammatory state, the levels of anti-inflammatory cytokines IL-4, IL-5, and IL-10 decline with age [11]. Therefore, age-related disruption of the pro- and anti-inflammatory cytokine homeostasis shifts the balance towards a chronic state of low-grade inflammation that contributes to age-related pathologies.

Increased age is one of the primary risk factors for most types of cancer, with the majority of newly diagnosed cases occurring in individuals over 65 years of age [12]. Multiple epidemiological studies have shown a correlation between chronic inflammation associated with “inflamm-aging” and increased incidence of cancer [13], [14]. Sustained inflammation disrupts tissue integrity and can drive tumorigenesis by creating a microenvironment that provides a selective advantage to the cells with acquired carcinogenic mutations, and thus, not requiring normal tissue homeostasis for survival [15]. However, the microenvironment of established tumors has been shown to be skewed toward an anti-inflammatory, T_H_2, response with an increase in IL-4 and IL-5 levels [16], [17]. Therefore, it is conceivable that the state of “inflamm-aging” creates a permissive microenvironment allowing tumor growth. Subsequently, the growing tumor induces a switch from the pro-inflammatory, T_H_1-like state to the tumor protective anti-inflammatory, T_H_2-like environment.

Multiple myeloma (MM) is a hematological malignancy with plasma cells comprising the bulk of the malignant clone. While recent clinical advances have improved outcomes, patients who enter clinical remission eventually relapse and become refractory to treatment [18]. The median age at diagnosis of MM is 70 years with 63% of cases attributed to individuals over the age of 65 suggesting that pathogenesis in MM correlates with aging [19]. MM is characterized by elevated serum monoclonal immunoglobulin produced by the excess of the malignant plasma cells, resulting in neuropathy, immunosuppression, and renal failure [20]. Additional symptoms of MM include bone pain and pathologic fractures that result from osteoclast-mediated bone destruction and present as lytic bone lesions that do not heal even after patients enter clinical remission [21]. Persistence of bone lesions throughout the course of the disease and the inevitable relapse of MM suggest that the systemic homeostasis is disrupted as a result of the disease process is not restored by currently available treatments and likely contributes to the eventual relapse.

Active MM is promoted by local bone marrow microenvironment, and systemic cytokines have been implicated in the survival and expansion of the MM clone [22]. Cells within the MM niche, i.e. bone marrow stromal cells, produce large quantities of IL-6, IL-7, IL-8, and TGFβ which regulate the growth and survival of malignant cells, maintain the cytokine feedback loops, and sustain the pro-tumorigenic environment [23], [24], [25]. Autocrine production of IL-15 has been shown to contribute to MM cell survival [26]. However, not all cytokines have tumor promoting effects. Interleukin 1 receptor antagonist (IL-1ra) has been shown to block proliferation of MM cells by blocking prostaglandin E2-mediated secretion of IL-6 [27]. These multiple alterations of the cytokine milieu within the bone marrow of MM patients lead to the suppression of the adaptive immunity; thus, bacterial infections remain the leading cause of death from MM [28].

To understand the biology behind the inevitable relapse of MM we explored the systemic milieu during clinical remission instead of during active disease as pursued by most studies. We compared the expression levels of systemic cytokines between normal donors (<60 years old), age-matched normal donors (>60 years old), MM patients with newly diagnosed MM or newly diagnosed relapse, patients undergoing treatment, and patients in clinical remission. Surprisingly, our study revealed that systemic homeostasis is not restored when patients enter remission and that the cytokine profiles do not change throughout the course of the disease. We propose that therapies aimed at shrinking the tumor do not restore homeostasis within the tissue; thus, the elements of the tumor-associated microenvironment remain post therapy sustaining the production of pro-tumorigenic factors that ultimately induce a relapse.

## Methods

### Ethics statement

Blood samples were collected after approval from Purdue University and University of Alberta Institutional Research Boards and after written informed consent in accordance with the Declaration of Helsinki.

### Study participants

The test population consisted of 8 patients with monoclonal gammopathy of undetermined significance (MGUS) and 55 patients with MM enrolled at the Cross Cancer Institute of the University of Alberta (Table 1). Healthy controls were broken into 2 groups: 77 anonymous discard blood samples taken from the City of Hope Blood Bank and 100 anonymous discard blood samples taken from the University of Alberta Hospital Emergency room. All control samples were screened to remove those from individuals who presented with malignancies or infections, as well as to exclude any samples with elevated white blood cell counts.

**Table 1:** Demographics of plasma donor populations.

	*N1* 1	*N2* 2	*MGUS* 3	*MM* 4
**# of samples**	77	100	8	55
* Male*	29	64	3	32
* Female*	48	36	5	23
**Age**				
* Mean*	46+/−10	73 +/−8	75+/−10	69+/−10
* Range*	19–60	61–94	57–86	38–84
**Disease stage**				
* Active disease*				12
* Treatment*				27
* Remission*				15
**Treatment**				
* Biological* #				13
* Conventional* ##				14

1 N1, Normal 1; <60 years old.

2 N2, Normal 2; >60 years old.

3 MGUS; monoclonal gammopathy of undetermined significance.

4 MM; multiple myeloma.

# Biological: bortezomib, lenalidomide.

## Conventional: melphalan, dexamethasone, prednisone.

### Plasma samples

Blood samples obtained from patients and normal controls were assigned an alphanumeric code and treated anonymously throughout this study. Blood was collected in heparin (green-top) tubes, centrifuged at 1,500 rpm for 10 min, aliquoted into 500 μl fractions, and frozen at −80°C for future use. To avoid any artifacts due to freeze-thaw, a single aliquot of plasma was used for each analysis; plasma was never re-frozen. All control samples were processed within 24 hrs after blood draw and MM and MGUS samples were processed and frozen within 1–3 hrs; samples were refrigerated prior to processing.

### Cytokine multiplex analysis

Plasma samples were analyzed for 25 cytokines using the Human Cytokine Twenty-Five-Plex Antibody Bead Kit (Biosource International, Camarillo, CA) per the manufacturer's protocol [29]. Briefly, Biosource's multiplex beads solution were vortexed for 20 s and 25 μl was added to each well and washed twice with wash buffer. The samples were diluted 1∶2 with assay diluent and loaded onto Millipore Multiscreen BV 96-well filter plate; 50 μl of incubation buffer had been added previously to each well. Serial dilutions of cytokine standards were prepared in parallel and added to the plate. Samples were incubated on a plate shaker at 600 revolutions/min in the dark at room temperature for 2 hours. The plate was applied to a Millipore Multiscreen Vacuum Manifold and washed three times with 200 μl of wash buffer. Biotinylated Anti-Human Multi-Cytokine Reporter (100 μl) was added to each well. The plate was incubated on a plate shaker for 1 hour. After three washes with 200 μl of wash buffer, 100 μl of streptavidin-phycoerythrin was added to each well followed by incubation on a plate shaker for 30 min. The plate was applied to the vacuum manifold, washed three times, and each well was resuspended in 100 μl wash buffer and shaken for 1min. The assay plate was transferred to the Bio-Plex Luminex 100 XYP (Bio-Rad Laboratories, Inc., Hercules, CA) instrument for analysis. Cytokine concentrations were calculated using Bio-Plex Manager 3.0 software with a five parameter curve-fitting algorithm applied for standard curve calculations.

### Enzyme-linked immunosorbent assay (ELISA)

Levels of IL-3, IL-6, IL-7, IL-15, IL-17, and TGFβ2 were measured using Quantikine ELISA kits (R&D Systems) per manufacturer's instructions. Plasma samples [N2, n = 22; MGUS n = 8; MM; n = 40] were diluted per kit protocol, with the exception of IL-15 kit where samples were diluted 1∶1. All MGUS samples were included in this analysis, while normal and MM samples were randomly chosen from our total set of plasma samples. A Thermo Multiskan Ascent plate reader was used to read the plates according to the manufacturer's instructions. Thermo Ascent and Graphpad Prism 5.0 software packages were used for data analysis.

#### LC/MS/MS analysis

Following a tryptic digestion of the plasma samples according to a method of J. E. Hale [30] peptides were separated on a nano LC/MS system which included an Agilent 1100 Series Capillary and Nanoflow pumps, Micro-Well Plate Sampler with Thermostat, and Chip Cube MS interface on the Agilent 6330 XCT ion trap mass spectrometer (Agilent Technologies, Santa Clara, CA). The peptides were loaded at 3 µl/min on an Agilent chip containing a 40 nl enrichment column packed with Zorbax 300SB-C18 5 µm material. The enrichment column was switched into the nano-flow path after 5 min, and peptides were separated with an analytical column (0.75 μm×150 mm, 5 μm) packed with C18 reverse phase ZORBAX 300SB-C18 5 µm material at a flow rate of 0.3 μl/min. The chip is coupled to the electrospray ionization (ESI) source of the ion trap mass spectrometer. The peptides were eluted from the column using an acetonitrile/0.01% trifluoroacetic acid (mobile phase B) linear gradient. For the first 5 min the column was equilibrated with 95% water/0.01% trifluoroacetic acid (mobile phase A) followed by a linear gradient of 5% B to 35% B over 55 min. The column was washed with 100% of ACN/0.01% TFA and equilibrated with 95% of H2O/0.01%TFA before the next sample was injected. A blank injection was run between samples to avoid carryover.

The XCT ion trap mass spectrometer was operated in positive MS mode and data-dependent positive acquisition MS/MS mode. MS spectra were collected for every sample in the study using an acquisition mass range from 350–2000 mass to charge ratio (m/z), a maximum accumulation time of 0.15 sec, and a scan speed of 8,100 m/z per second. In order to compile a protein database, random samples were run in MS/MS mode where the three most abundant molecular ions were selected and fragmented (0.5 min active exclusion; 2+ ions preferred) by collision induced dissociation (CID).

Database search analysis was done using Spectrum Mill MS Proteomics Workbench by Agilent (Rev A.03.03.084 SR4). The Agilent. D raw files generated were converted to. mzxml files and searched against the NCBI protein database, species homo sapiens. Cysteine alkylation by iodoethanol was selected in the search set up. Proteins were identified based on the score and percent scored peak intensity (SPI%) values and those with a minimum of 3 peptide hits with p-values <0.05 were considered significant.

### Statistical analysis

Expression levels of IL-3 and TGFβ2 were presented as mean ± s.e.m. Statistical significance for ELISA data and expression levels of IL-17 at different stages of MM were analyzed by a one-way ANOVA with Tuckey's post-test using GraphPad Prism software (version 5.0, GraphPad Software, San Diego, CA) with p-value <0.05 considered significant.

Data processing and differential expression analysis of Luminex data were performed with the Bioconductor and R software package. Cytokine expression was transformed to the log_2_ scale and quantile-normalized. Box-and-whisker plots were used to visualize the distribution of intensities across subjects. Linear models were fit for each cytokine using Limma to test for differential expression for pre-specified contrasts [31]. P-values for each contrast were obtained for each cytokine and adjusted for multiple comparisons using the False Discovery Rate (FDR) using the Benjamini-Hochberg procedure. Hierarchical clustering was performed using R software package.

Cytokines with strong evidence of differential expression (>1.25 fold change either up or down) between MM and N2, between MGUS and N2, and between MM and MGUS were selected according to those cytokines having an adjusted p-value <0.05, and these were used in a linear discriminant analysis (LDA) to evaluate their predictive potential. To assess accuracy, leave-one-out cross-validation was used, and the cross-validated accuracy rates are reported. Due to the small sample size of the MGUS group, classification was also performed on the remaining three groups to assess accuracy when only MM, N1, and N2 were considered, and the leave-one-out cross-validated accuracy rates are reported. Samples collected at first diagnosis or relapse were grouped together during analysis and will be further referred to as “active disease”.

## Results

### Cytokine expression exhibits age-related changes in favor of a pro-inflammatory environment

**Figure 1:**
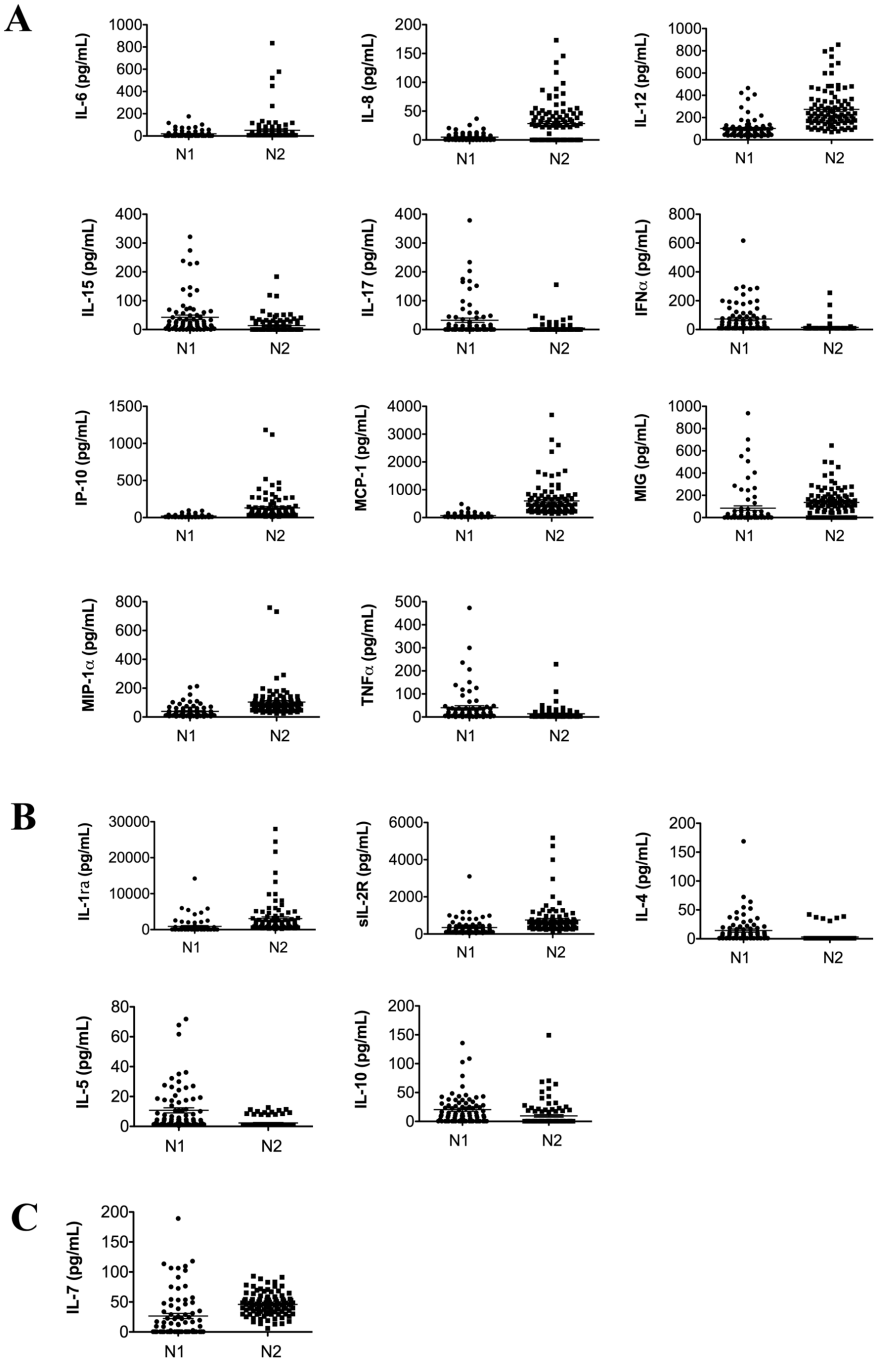
Expression of systemic cytokines in healthy donors changes with age. Expression of 25 cytokines was measured utilizing multiplex technology of the Bio-Plex Luminex 100 XYP in the plasma of healthy adults, N1 and N2, ages 46±10 and 73±8 respectively. Differential expression of systemic cytokines between N1 and N2 populations of healthy donors (fold change >1.25 with p-values <0.05; exact values for all fold-changes and p-values are in Table 3). (**A**) pro-inflammatory cytokines (**B**) anti-inflammatory cytokines, (**C**) lymphocyte growth factor.

In order to understand how systemic cytokines influence the progression of multiple myeloma, a disease of the older adults, we first examined how cytokine profiles change in healthy individuals with age. Blood samples collected from healthy donors where subdivided into 2 groups: N1 (mean age 46 years) and N2 (mean age 75 years) (Table 1), and the plasma levels of 25 cytokines were measured. We observed an increase in the expression of pro- and a decrease in the expression of anti-inflammatory cytokines (Figure 1, Tables 2 and 3). Out of 11 pro-inflammatory cytokines and chemokines with the significantly altered expression between N1 and N2 populations, 7 were elevated (IL-6, IL-8, IL-12, IP-10, MCP-1, MIG, and MIP-1α) and 4 decreased (IL-15, IL-17, IFNα) (Figure 1A). The expression levels of 3 out of 5 anti-inflammatory cytokines were decreased in N2 population compared to N1 (IL-4, IL-5, IL-10) (Figure 1B). We also observed that the N2 cohort exhibited an elevated expression of IL-7, a lymphocyte growth factor, (Figure 1C) suggesting that the elevation in pro-inflammatory cytokines may be due to an increase in IL-7 induced differentiation and proliferation of lymphocytes [32].

**Table 2:** Cytokine expression levels in N1, N2, MGUS, and MM cohorts.

*Cytokine/chemokine*	*N1 (AVG*±*SEM)*	*N2 (AVG*±*SEM)*	*MGUS (AVG*±*SEM)*	*MM (AVG*±*SEM)*
**IL-1**β	45.63±6.09	149.8±56.5	17.88±4.63	80.71±30.89
**IL-1ra** #	902.4±233.1	3062±468.5	245.6±39.28	904.7±313.9
**IL-2**	12.51±2.01	59.67±35.85	2.04±0.92	26.24±13.12
**sIL-2R** #	347.1±48.08	751.1±80.28	1270±441.5	1099±113.2
**IL-3**	ND	29.24±9.81	0.93±0.44	6.83±2.01
**IL-4** #	14.19±2.73	3.037±0.86	6.63±0.29	10.02±0.56
**IL-5**	10.74±1.69	2.29±0.30	1.11±1.06E-7	1.72±0.43
**IL-6**	19.28±3.51	50.9±12.19	24±13.7	16.61±3.03
**IL-7** #	26.62±4.35	45.97±1.83	65.22±4.66	59.01±2.33
**IL-8**	4.89±0.73	28.46±3.45	30.06±4.38	41.46±9.26
**IL-10** #	20.39±2.93	9.53±2.19	15.14±3.75	18.51±1.56
**IL-12**	102.1±10.13	273.3±17.6	145.1±23.53	282.2±58.92
**IL-13** #	27.23±4.97	25.03±2.59	0.40±0.001	0.51±0.12
**IL-15**	42.58±7.61	13.45±2.86	9.93±0.55	26.17±9.05
**IL-17** #	32.29±7.54	4.68±1.77	0.85±0.35	27.46±4.53
**Eotaxin** #	64.37±5.06	90.04±5.08	122.6±11.9	131.9±8.43
**GM-CSF** #	47.46±8.97	20.78±2.29	0.4±0.001	4.0±1.19
**IFN**α	72.94±11.45	14.92±0.05	11.03±1.75	17.51±2.65
**IFN**γ #	32.62±5.90	28.51±3.45	1.92±0.29	3.77±0.17
**IP-10**	20.26±1.98	130.9±17.98	132.9±24.52	179.9±25.35
**MCP-1** #	72.76±8.14	597.2±58.78	661.4±127.1	867.7±88.22
**MIG** #	84.75±20.40	136.2±12.36	85.51±33.36	76.16±8.59
**MIP-1**α	38.93±4.77	103.6±0.34	44.62±3.45	129.0±32.97
**MIP-1**β	370.5±83.20	203.1±52.2	219.8±160.0	173.8±49.67
**RANTES**	153529±31640	256198±15016	125789±44631	182050±21161
**TGF**β**2**	ND	1.51±0.52	0.54±0.28	3.07±0.57
**TNF**α	40.41±8.44	13.85±2.62	4.68±2.51	11.32±4.6

# cytokines included in LDA.

ND, not determined.

**Table 3:** Fold change in cytokine expression levels.

*Cytokine/chemokine*	*Fold change (N1/N2)*	*p-value (N1 vs. N2)*	*Fold change (MGUS/N2)*	*p-value (MGUS vs. N2)*	*Fold change (MM/N2)*	*p-value (MM vs. N2)*	*Fold change (MGUS/MM)*	*p-value (MGUS vs. MM)*
**IL-1**β	0.30	>0.05	0.12	>0.05	0.54	>0.05	0.22	>0.05
**IL-1ra**	0.29	0.0002	0.08	0.0018	0.30	<0.0001	0.27	>0.05
**IL-2**	0.21	>0.05	0.03	>0.05	0.44	>0.05	0.08	>0.05
**sIL-2R**	0.46	<0.0001	1.69	>0.05	1.46	0.0002	1.16	>0.05
**IL-3**	ND	ND	0.03	0.01	0.23	0.0064	0.14	>0.05
**IL-4**	4.67	<0.0001	2.18	0.0012	3.30	<0.0001	0.66	>0.05
**IL-5**	4.69	<0.0001	0.48	>0.05	0.75	>0.05	0.65	>0.05
**IL-6**	0.38	0.0276	0.47	>0.05	0.33	>0.05	1.44	>0.05
**IL-7**	0.58	<0.0001	1.42	0.0014	1.28	<0.0001	1.11	>0.05
**IL-8**	0.17	<0.0001	1.06	>0.05	1.46	>0.05	0.73	>0.05
**IL-10**	2.14	0.0028	1.59	>0.05	1.94	<0.0001	0.82	>0.05
**IL-12**	0.37	<0.0001	0.53	>0.05	1.03	>0.05	0.51	>0.05
**IL-13**	1.09	>0.05	0.02	<0.0001	0.02	<0.0001	0.78	>0.05
**IL-15**	3.17	0.0001	0.74	>0.05	1.95	>0.05	0.38	>0.05
**IL-17**	6.90	<0.0001	0.18	>0.05	5.87	<0.0001	0.03	0.0049
**Eotaxin**	0.71	0.0006	1.36	0.0354	1.46	0.0083	0.93	>0.05
**GM-CSF**	2.28	>0.05	0.02	<0.0001	0.19	<0.0001	0.10	>0.05
**IFN**α	4.89	<0.0001	0.74	>0.05	1.17	>0.05	0.63	>0.05
**IFN**γ	1.14	>0.05	0.07	<0.0001	0.13	<0.0001	0.51	>0.05
**IP-10**	0.15	<0.0001	1.02	>0.05	1.37	>0.05	0.74	>0.05
**MCP-1**	0.12	<0.0001	1.11	>0.05	1.45	0.0027	0.76	>0.05
**MIG**	0.62	0.0251	0.63	>0.05	0.56	0.0021	1.12	>0.05
**MIP-1**α	0.38	<0.0001	0.43	>0.05	1.25	>0.05	0.35	>0.05
**MIP-1**β	1.82	>0.05	1.08	>0.05	0.86	>0.05	1.26	>0.05
**RANTES**	0.60	>0.05	0.49	>0.05	0.71	>0.05	0.69	>0.05
**TGF**β**2**	ND	ND	0.36	>0.05	2.03	>0.05	0.18	>0.05
**TNF**α	2.92	0.0011	0.34	>0.05	0.82	>0.05	0.41	>0.05

**Figure 2:**
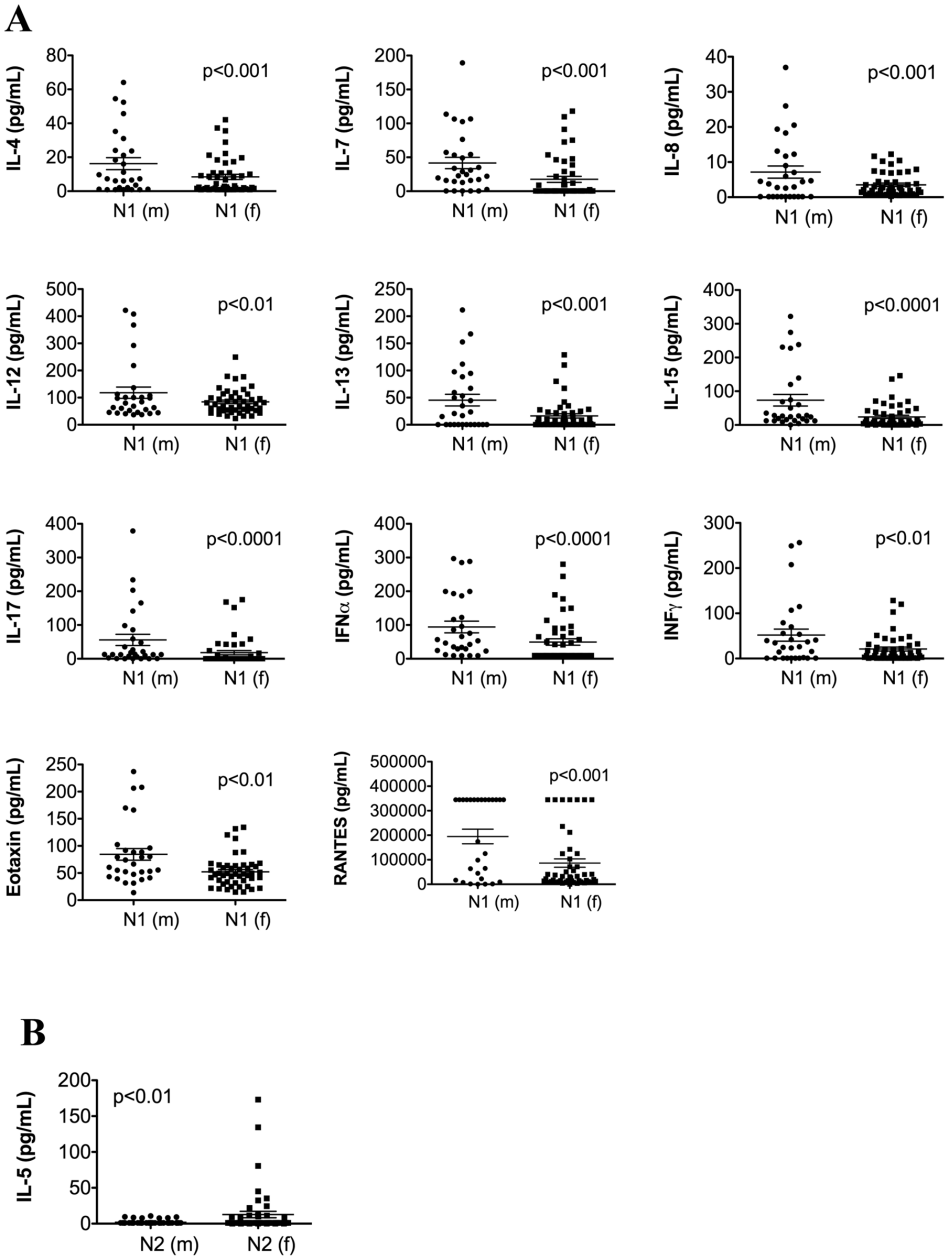
Multiple cytokines are elevated in healthy males compared to females: the difference that is lost with age. Gender differences in the expression of systemic cytokines in each population of healthy donors (fold change >1.25 with p-values <0.05). (**A**) Male versus female expression of cytokines in N1 population, (**B**) Male versus female expression of cytokines in N2 population, (**C**) Gender differences in cytokines differentially expressed between N1 and N2 cohorts.

Next, we examined gender-related cytokine changes as a function of age. Among the N1 population 11 cytokines and chemokines (pro-inflammatory: IL-8, IL-12, IL-15, IL-17, IFNα, IFNγ, RANTES; anti-inflammatory: IL-4, IL-13, eotaxin; and lymphocyte growth factor: IL-7) were reduced in females compared to males, creating a net pro-inflammatory environment in women (Figure 2A). However, only IL-5 was differentially expressed in the N2 population with female donors exhibiting an elevated expression (Figure 2B). When we compared the cytokine expression separately in males and females between N1 and N2 populations (i.e. N1(m) vs. N2(m) and N1(f) vs. N2(f)), we observed the same differences as those seen when both genders were included in the analysis (Figure 1). Finally, when we evaluated the impact of gender on the levels of cytokines differentially expressed between N1 and N2 (i.e. [(N1 vs. N2)(m)] vs. [(N1 vs. N2)(f)]), we found that 7 out of 17 cytokines (pro-inflammatory: IL-6, IL-8, IL-17, IP-10; anti-inflammatory: IL-4; and lymphocyte growth factor: IL-7) that were statistically different between N1 and N2 were also differentially modulated by gender. Interleukin-7, IL-15, and IL-17 were increased in the male population, while IL-4, IL-6, IL-8, and IP-10 were increased in the female group (Table 4).

**Table 4:** Gender differences in cytokines differentially expressed between N1 and N2.

*Cytokine/chemokine*	*Fold change (F vs. M)*	*p-value (F vs. M)*
**IL-4**	2.148	0.015
**IL-6**	2.854	0.011
**IL-7**	0.34	0.010
**IL-8**	6.506	0.001
**IL-15**	0.245	0.028
**IL-17**	0.196	0.011
**IP-10**	3.191	0.023

### Humoral immunity of patients with multiple myeloma is shifted towards an anti-inflammatory phenotype

**Figure 3:**
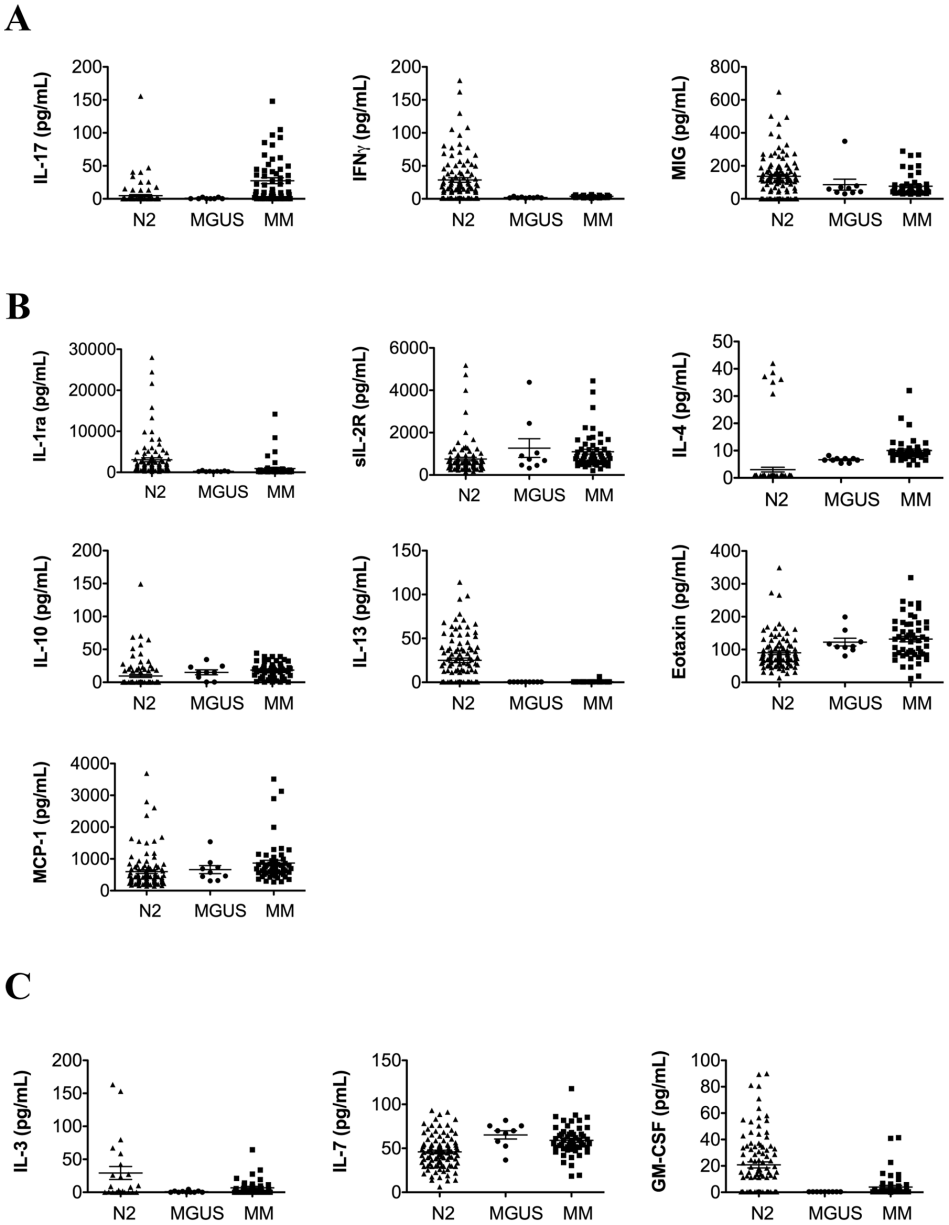
Patients with multiple myeloma present with a systemic cytokine profile consistent with an anti-inflammatory environment. Differential expression of cytokines between N2, MGUS, and MM populations (fold change >1.25 with p-values <0.05; exact values for all fold-changes and p-values are in Table 3). MGUS* denotes those cytokines with significant differences in expression between MGUS and MM; MGUS** denotes those cytokines with significant differences in expression between MGUS and N2; (**A**) pro-inflammatory cytokines (**B**) anti-inflammatory cytokines, (**C**) lymphocyte growth factors.

Next, we evaluated the alternations in systemic cytokines as a function of progression from the healthy state through the pre-malignant condition, MGUS, to full blown malignancy, MM. Since we observed significant age-related differences in cytokine expression between N1 and N2 populations, for the next set of analyses we used the N2 cohort of plasma samples from healthy donors that were age-matched to those from MGUS and MM patients. Overall, 13 cytokines were differentially expressed between N2 and MM groups. Patients with MM exhibited a shift toward an anti-inflammatory and pro-tumorigenic phenotype marked by a significant decrease in the expression of 2 out of 3 pro-inflammatory cytokines (IFNγ and MIG) (Figure 3A) and a significant elevation in the expression of 5 out of 7 anti-inflammatory cytokines (sIL-2R, IL-4, IL-10, eotaxin, MCP-1) (Figure 3B). The expression levels of the lymphocyte growth factors were variable with decreases in IL-3 and GM-CSF and an increase in IL-7 in MM patients compared to healthy controls (Figure 3C). Consistent with other studies that show an elevation of IL-6 in the bone marrow plasma, but not peripheral blood plasma since the bone marrow sinus is separated from the peripheral circulation, systemic levels of IL-6 were not elevated in our study.

Analysis of differential expression of cytokines in MM samples compared to healthy age-matched controls did not reveal any additional statistically significant differences. Similarly to the lack of gender-bias in the cytokine expression in the N2 population, comparison of cytokine expression levels between male and female patients within the MM cohort did not produce statistically significant differences. When we compared the expression of cytokines and chemokines between healthy males and those with MM or healthy females and women with MM (i.e. N2(m) vs. MM(m) and N2(f) vs. MM(f)), we did not detect any gender-specific differences; both male and female populations exhibited changes consistent with those of the combined population (Figure 3).

The hallmark of MM is the overexpression of monoclonal immunoglobulin resulting in suppression of production of polyclonal antibodies, and thus a suppression of immune responses. In addition to antibodies, cytokines and complement constitute two other components of the humoral immunity. The data above demonstrates that the cytokine profile of patients with MM is skewed toward an anti-inflammatory, and thus, immunosuppressive phenotype. Therefore, to assess all aspects of the humoral immunity we evaluated the levels of plasma complement components in patients with MM compared to N2 age-matched controls (Table 5). Using tandem LC/MS/MS we established that the levels of 2 inhibitors of the complement pathway, factor H and C1i, were elevated in plasma of MM patients with the mean increase of 6.67 and 10-times respectively. While the expression of complement component C4 and precursors of factor B and C3 were also elevated in patients with MM (5.7, 5.26, and 7.14-times respectively), there was a 9.74-fold reduction in the levels of the activated complement component C3b. Since C3b is central to the formation of the C3 and C5 convertases, and thus, is essential for the effector functions of the complement pathway, a decrease in C3b contributes to the immunosuppressive state of patients with MM.

**Table 5:** Analysis of the complement pathway components.

*Protein*	*Access. #*	*Peptides*	*Fold change (MM/N2)*	*p-value*
**complement factor**	67782358	(K)YGQTIRPICLPCTEGTTR(A)	4.00	8.68E–03
**B preproprotein**		(R)DFHINLFQVLPWLK(E)	5.26	9.22E–03
		(K)YGQTIRPICLPCTEGTTR(A)	3.85	9.64E–03
		(R)YGLVTYATYPK(I)	10.00	1.00E–02
		(R)DFHINLFQVLPWLK(E)	6.67	1.11E–02
**complement factor H**	758073	(R)NTEILTGSWSDQTYPEGTQAIYK(C)	9.09	8.62E–03
	31965	(R)SITCIHGVWTQLPQCVAIDK(L)	4.76	9.79E–03
complement factor H-related 3	54792787	(R)RPYFPVAVGK(Y)	7.69	1.02E–02
**complement component 1 inhibitor precursor**	73858568	(K)HRLEDMEQALSPSVFK(A)	10.00	7.69E–03
		(K)TRmEPFHFKNSVIK(V)	10.00	1.08E–02
		(K)GVTSVSQIFHSPDLAIR(D)	10.00	1.20E–02
**complement component C3b**	284052	(K)VYAYYNLEESCTR(L)	0.09	1.09E–02
complement component C3	179665	(K)RIPIEDGSGEVVLSR(K)	0.10	9.17E–03
PREDICTED: similar to complement component 3	169214179	(R)SEETKENEGFTVTAEGK(G)	0.12	9.03E–03
**complement component**	115298678	(K)DFDFVPPVVR(W)	10.00	9.02E–03
**3 precursor**		(K)EYVLPSFEVIVEPTEK(F)	6.25	1.06E–02
		(K)LSINTHPSQKPLSITVR(T)	10.00	9.84E–03
		(K)QDSLSSQNQLGVLPLSWDIPELVNMGQWK(I)	7.14	8.33E–03
		(K)SGQSEDRQPVPGQQMTLK(I)	4.55	6.06E–07
		(R)EPGQDLVVLPLSITTDFIPSFR(L)	7.14	9.60E–03
		(R)ILLQGTPVAQMTEDAVDAER(L)	4.17	9.95E–03
		(R)IPIEDGSGEVVLSR(K)	10.00	1.02E–02
		(R)SGIPIVTSPYQIHFTK(T)	7.14	1.01E–02
		(R)TELRPGETLNVNFLLR(M)	8.33	9.65E–03
		(R)TVMVNIENPEGIPVK(Q)	10.00	7.17E–03
		(R)VPVAVQGEDTVQSLTQGDGVAK(L)	5.56	8.85E–03
		(K)TKDKITHFTILVLSK(G)	10.00	9.58E–03
**RecName: Full = C4-A**	81175238	(K)VGLSGMAIADVTLLSGFHALR(A)	8.33	1.14E–02
**Complement precuresor**		(R)DFALLSLQVPLK(D)	6.25	1.13E–02
		(R)EMSGSPASGIPVK(V)	7.14	1.01E–02
		(R)HLVPGAPFLLQALVR(E)	5.00	1.04E–02
		(R)RGHLFLQTDQPIYNPGQR(V)	5.26	1.07E–02
		(R)STQDTVIALDALSAYWIASHTTEER(G)	3.85	9.80E–03
		(R)YVSHFETEGPHVLLYFDSVPTSR(E)	7.14	1.01E–02
		(R)YIYGKPVQGVAYVR(F)	10.00	1.02E–02
		(R)GPEVQLVAHSPWLK(D)	10.00	1.07E–02
		(K)VLSLAQEQVGGSPEK(L)	10.00	7.61E–03
		(R)GPEVQLVAHSPWLK(D)	3.85	1.00E–02
		(R)KADGSYAAWLSR(D)	3.03	1.12E–02

### Cytokine homeostasis is not restored in response to treatment or during remission

**Figure 4:**
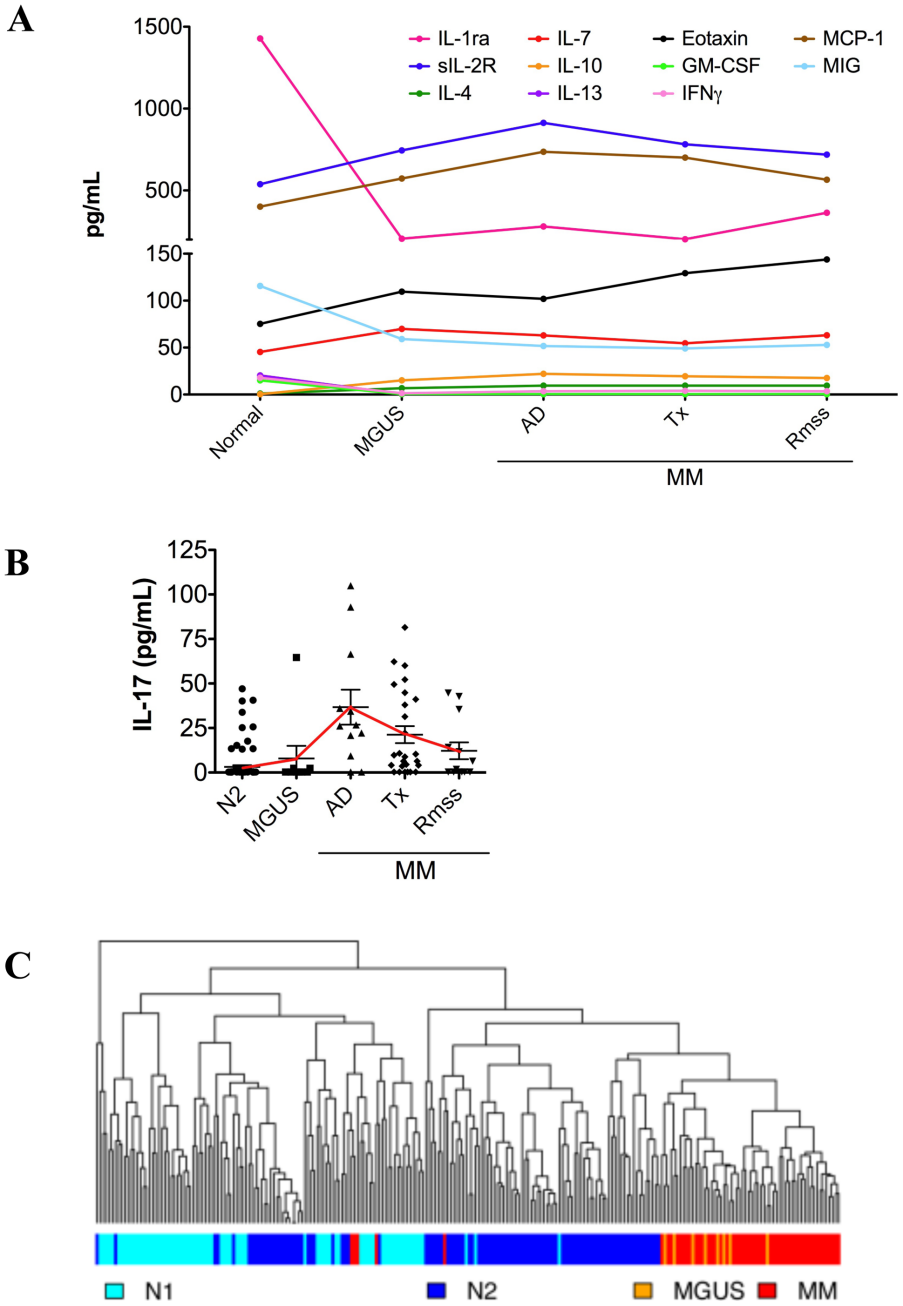
Cytokine profile of patients entering remission from multiple myeloma is not restored to homeostasis. (**A**) Levels of cytokines differentially expressed between N2 and MM were evaluated as a function of the disease stage (i.e. active disease (AD), treatment (Tx), and remission (Rmss)). Median expression values for each cytokine are plotted. All cytokines levels were statistically different (p<0.01) between N2 and MM, but not between the stages of MM (p>0.05). With the exception of sIL-2R, MCP-1, and MIG, all cytokines were differentially expressed between N2 and MGUS (<0.03). (**B**) Expression of IL-17, the only cytokine expression of which is restored after treatment, for each stage of MM. The solid line denotes the trend of IL-17 expression over the course of the disease. Statistical significance: N2 vs. MGUS, p>0.05; N2 vs. active disease, p<0.0001; N2 vs. treatment, p<0.0001; N2 vs. remission, p>0.05. (**C**) Dendogram depicting the results of hierarchical clustering of all plasma donors based on their placement into N1, N2, MGUS, and MM cohorts. The colored bar shows the placement of each donor within the study populations (N1, light blue; N2, dark blue; MGUS, orange; MM, red).

Since the homeostasis of systemic cytokines is disrupted in MM, we wanted to determine whether the cytokine profile returns to normal once treatment is administered and a patient enters remission. The set of samples from MM patients were grouped into those with active disease (n = 12), undergoing treatment (n = 27), and in clinical remission (n = 15). At the time of blood draw, patients in the active disease group were either diagnosed with primary MM or its relapse, those in the treatment category were undergoing treatment, and those in clinical remission were classified by the treating physician as in partial or complete remission. Our data showed that cytokine profiles did not change significantly over the course of MM and remained steady at the active disease levels throughout treatment and into remission phases (Figure 4A).

IL-17 was the only exception, with its levels rising during the active phase of MM, decreasing with treatment, and returning to normal in remission (Figure 4B). Interleukin-17 was significantly elevated in the plasma of MM patients with active disease and during treatment (p<0.0001 compared to N2). However, its levels were not significantly different when comparing MM patients in remission to healthy controls (p>0.05 remission compared to N2). These findings suggest that the levels of IL-17 return to normal during remission from MM.

Finally, we wanted to assess whether the type of treatment received by MM patients affects the systemic cytokine profiles. Patients undergoing treatment at the time of blood collection were divided into two groups: 1) those receiving a regimen containing conventional drugs either as single agents or as combination therapy (i.e. melphalan, dexamethasone, and prednisone) (n = 14) and 2) those on combination therapy that included novel biological agents bortezomib or lenalidomide together with the conventional chemotherapeutic agents (n = 13). We did not detect any differences in the cytokine expression as a function of the treatment regimen.

The expression of systemic cytokines in patients with MGUS closely followed the patterns seen in MM patients (Figure 3A–C). However, of 13 cytokines differentially expressed between N2 and MM, only 8 were differentially expressed between N2 and MGUS. Levels of IL-1ra, IL-3, IL-13, GM-CSF, and IFNγ were reduced, while the expression of IL-4, IL-7, and eotaxin was elevated in the plasma of patients with MGUS (Figure 3A–C). Interleukin-17 was the only cytokine differentially expressed between MGUS and MM, but not between MGUS and N2 samples (Figures 3A and 4B).

### Differential expression of systemic cytokines can be used to classify individuals into healthy and MM categories

We wanted to evaluate whether cytokine profiles can accurately classify an individual as healthy or having MM. Linear discriminant analysis (LDA) was applied to evaluate the predictive potential of differentially expressed cytokines and chemokines (Table 2). The accuracy of the model was tested by performing leave-one-out cross-validation and the accuracy rates of the predictions are shown in Tables 6 and 7 (actual confusion matrix is shown in Tables 8 and 9). Using LDA we could efficiently classify healthy controls into N1 or N2 category with 0.22 and 0.07 error rates for classification into N1 and N2 populations respectively. Interestingly, LDA was more accurate in predicting that the sample belongs to the N2 group, likely due to the higher variance in cytokine levels within N1 population (9 out of 12 cytokines included in the model N1 had higher variance than N2).

**Table 6:** Accuracy rates of LDA classification for healthy, MGUS, and MM populations.

	Predicted:			
True:	MGUS	MM	N1	N2
**MGUS**	**0.25**	0.75	0.00	0.00
**MM**	0.09	**0.89**	0.02	0.00
**N1**	0.01	0.01	**0.88**	0.09
**N2**	0.00	0.02	0.05	**0.93**

**Table 7:** Accuracy rates of LDA classification for healthy and MM populations.

	Predicted:		
True:	MM	N1	N2
**MM**	**0.98**	0.02	0.00
**N1**	0.01	**0.89**	0.09
**N2**	0.02	0.05	**0.93**

**Table 8:** Confusion matrix for classification of healthy, MGUS and MM populations.

	Predicted:			
True:	MGUS	MM	N1	N2
**MGUS**	**2**	6	0	0
**MM**	5	**49**	1	0
**N1**	1	1	**68**	7
**N2**	0	2	2	**96**

**Table 9:** Confusion matrix for classification of healthy and MM populations.

	Predicted:		
True:	MM	N1	N2
**MM**	**54**	1	0
**N1**	1	**70**	6
**N2**	2	2	**96**

LDA could not accurately differentiate (0.75 error rate) MGUS from MM identifying 2 MGUS samples correctly and but placing the remaining 6 in the MM category (Table 8). MM could be predicted with 89% accuracy (Tables 6 and 8), and hierarchical clustering of all samples showed that MM segregated very well from N1 and N2 populations, but not from MGUS (Figure 4C). Therefore, we re-ran LDA with only N1, N2, and MM cohorts. When MGUS samples were dropped, the accuracy of prediction increased to 98% with only 1 MM patient incorrectly classified as N1 (Tables 7 and 9). When we initiated the study we expected the cytokine profiles of patients in remission from MM to return to pre-disease levels, however, as shown in Figure 4A, once the disease was initiated, the cytokine levels were not restored to normal levels even post therapy. Consistent with this finding, LDA placed 14 out of 15 patients in remission in MM category, with only one identified as N1. Taken together, our results show that the systemic environment of patients with MM, even those in clinical remission, is skewed toward an anti-inflammatory, and thus, pro-tumorigenic phenotype. The finding that the cytokine profile of MM patients in clinical remission are not restored to pre-disease homeostasis suggests that such an environment may support minimal residual disease and contribute to the inevitable relapse of MM patients.

## Discussion

To paint a comprehensive picture of the dynamic changes in the cytokine milieu throughout the lifetime of an individual, we performed multiplex cytokine profiling. We evaluated the shifts in cytokine homeostasis during active disease, treatment, and remission phases of MM. The baseline for each cytokine was set to its level in the N2 population that was age-matched to the MM cohort. Moreover, we extended the study of the systemic environment in MM to evaluation of the complement pathway, which together with cytokines and antibodies comprises the humoral immune system of the human.

Consistent with the concept of “inflamm-aging” [4], our comprehensive analysis revealed a shift toward a pro-inflammatory environment in individuals over 60 years old. We observed an increase in the expression of a number of pro-inflammatory cytokines and chemokines and a decrease in levels of major anti-inflammatory cytokines: IL-4, IL-5, and IL-10. During an infection, pro-inflammatory molecules such as IL-6, IL-12, and IP-10 are required to stimulate and maintain both adaptive and innate immune responses, and chemokines such as IL-8, MCP-1, MIG, and MIP-1α are responsible for recruitment of various inflammatory cells to the site of the infection. However, once the infection is cleared, the levels of inflammatory cytokines return to normal. Steadily accumulating evidence demonstrates that prolonged inflammation contributes to tumor formation [33]. Therefore, it is possible that the chronic state of inflammation observed in the individuals older than 60 years of age creates a permissive environment, and thus, promotes tumorigenesis.

A strong gender-bias was observed in the levels of systemic cytokines where the female population from the N1 cohort demonstrated a decrease across all cytokines differentially expressed between the male and female populations. Testosterone has been shown to stimulate the production of IL-10 and block the production of TNFα and IL-1βcreating a net anti-inflammatory environment [34], [35]. Lower levels of testosterone in women compared to men may be the basis for the observed gender bias toward a pro-inflammatory environment in women. Moreover, the decrease in levels of IL-4, a major cytokine required for an anti-inflammatory, TH2, response, in the female population suggests that the systemic environment in women is more prone to a pro-inflammatory, TH1, response, suggesting a possible mechanism for the higher incidence of TH1 driven autoimmune diseases in younger women [35]. However, within the N2 group of healthy donors, only IL-5 was differentially expressed between males and females, with the female population exhibiting significantly higher levels of IL-5. It is not entirely clear why the cytokine levels equalize with age across genders, but it is possible that the age-related changes in estrogen and testosterone may affect the cytokine production. Consistent with our findings of pro-inflammatory environment in N2 cohort, age-related decrease in testosterone expression in both men and women may release its block on TNFα and IL-1β expression, and thus, shift the cytokine homeostasis toward the pro-inflammatory environment defined by the concept of “inflamm-aging”.

**Figure 5:**
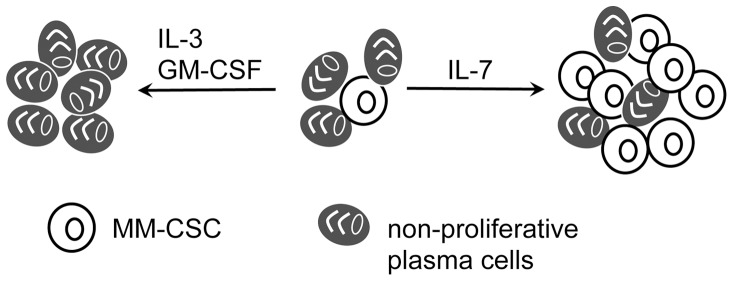
A model of possible regulation of MM-CSC by IL-3/GM-SCF and IL-7. Based on our observation that MM, but not normal, plasma supported the growth of the MM clone and maintained dormancy of MM-CSC [30], we speculate that IL-3 and GM-CSF induce differentiation of MM-CSC (unpublished), and have to be downregulated in patients to prevent the loss of the MM-CSC due to differentiation, and thus, contribute to maintaining the pool of MM-CSCs that can be activated to initiate a relapse. Conversely, since IL-7 stimulates proliferation of the B cells, its expression has to be maintained at higher levels to ensure that the B cell population of the multiple myeloma clone, and thus, the MM-CSC, persists through treatment and remission.

Chronic inflammation damages tissues and has been shown to generate a permissive, pro-tumorigenic microenvironment required for tumor development [33]. However, immunosurveillance, an attribute of the pro-inflammatory immunity, is responsible for the elimination of growing tumors, and is thus anti-tumorigenic once a tumor starts growing. Therefore, once a tumor is established, it sets up an environment skewed toward an anti-inflammatory phenotype that supports its growth and prevents recognition by the elements of immunosurveillance [36]. The N2 population of healthy adults over 60 years of age developed a milieu of systemic cytokines consistent with low-grade chronic inflammation, thus, creating an environment permissive for tumor development. In contrast, the cytokine profile of MM patients exhibited an anti-inflammatory phenotype. IFNγ has been shown to inhibit tumor growth [37], and we found both IFNγ and MIG, a monokine-induced by IFNγ, at lower levels in the plasma of patients with MM. Moreover, the expression of IL-4 and IL-10 stimulates a pro-tumorigenic response [14], [38], and these cytokines were expressed at higher levels in patients with MM compared to healthy controls. Interestingly, we also observed modulation in the expression of cytokines that regulate the function of the hematopoietic stem cells (IL-3, IL-7, and GM-CSF, also known as lymphocyte growth factors). Interleukin-3 and GM-CSF induce differentiation of hematopoietic stem cells [39], [40], and IL-7 is responsible for the commitment of the common lymphoid progenitor toward B cell lineage [41]. We propose that these 3 cytokines could play a role in the regulation of the MM cancer stem cell (MM-CSC), which has been proposed to be responsible for the almost certain relapse of MM patients [30], [42]. Reduction of IL-3 and GM-CSF expression in MM patients may be necessary to maintain the pool of the MM-CSC which otherwise may be lost due to IL-3/ GM-CSF mediated differentiation of these rare cells. Elevation of IL-7 may contribute to the expansion of the MM-CSC pool since IL-7 has been shown to be responsible for survival and proliferation of B cells [43] (Figure 5). Further supporting this model we recently observed an expansion of the MM-CSC population when IL-3 receptor is blocked with neutralizing antibodies or when recombinant human IL-7 is added to the culture (unpublished).

Consistent with the anti-inflammatory systemic environment in MM, there were multiple deviations in the expression of the components of both classical and alternative branches of the complement pathway. Levels of C1i and factor B, inhibitors of classical and alternative pathways respectively, were elevated 5–10-fold in plasma of MM patients, while C3b, the central active component required for the effector functions of both classical and alternative pathways, was reduced nearly 10-fold. Interestingly, C3, C4, and factor B precursors were elevated at least 5-fold, suggesting that there is an overproduction of the complement components, likely to compensate for a defect in their activation. Together with suppression of normal immunoglobulin production and cytokine profiles skewed toward an anti-inflammatory phenotype, the defect in the activation of complement components and the overexpression of the inhibitors of classical and alternative pathways contributes to the defective humoral immunity leading to immunosuppression seen in MM patients. Moreover, overproduction of factor B is one of the documented causes of renal failure [44], another symptom of MM.

We expected cytokine expression by MM patients in clinical remission to be restored to the levels roughly equal to those observed in healthy individuals, but surprisingly, the levels of all differentially expressed cytokines, with the exception of IL-17, did not change from active disease, through treatment, and into relapse phases of MM. This implies that the homeostasis of systemic factors is lost as early as the clinically pre-malignant MGUS stage. Furthermore, the lack of change in the production of the lymphocyte growth factors, IL-7 and GM-CSF, over the treatment and remission phases of MM suggests that aberrant cytokine expression may help sustain the MM-CSC population during remission, a speculation supported by previously identified MM-CSC growth conditions in 3-D cultures [30]. IL-17 was the only cytokine differentially expressed between MM and MGUS, but not MGUS and N2. The modulation of IL-17 over the course of MM is consistent with the recent finding that the numbers of IL-17 producing T_H_17 cells are elevated in patients with active disease, but are restored to normal with treatment [45].

Finally, we tried to build a predictive model that would classify patients into healthy, MGUS, and MM groups based on the levels of the differentially expressed cytokines. Interestingly, while our model performed well to correctly classify healthy and MM populations, it could not accurately differentiate between MGUS and MM samples. This may be due to a low number of MGUS samples included in this study or could have deeper roots in the biology of the disease where systemic aberrations occur early in malignant transformation. While MM and MGUS cannot be differentiated based on the cytokine expression, the profile of the differentially expressed cytokines seems to be a good predictor of MM. Therefore, a combination of differentially expressed cytokines could be used for early detection of clonal expansion that warrants further clinical evaluation to distinguish between patients with MGUS and those with MM. Taken together, our data demonstrates that with age the profile of systemic cytokines is shifted toward a pro-inflammatory phenotype supporting the concept of “inflamm-aging”, but the homeostasis becomes skewed toward a pro-tumorigenic, anti-inflammatory environment in MM. The failure to restore cytokine profiles to normal levels suggests that MM induces significant alterations in the prevalence of cytokine-producing cells. Such malignancy-sustaining microenvironment persists post treatment, and thus, is able to maintain aberrant cytokine milieu that likely contributes to the frequent relapse of MM.
